# Supplementary material for: Dimeric 1,4-Benzoquinone Derivatives with Cytotoxic Activities from the Marine-Derived Fungus Penicillium sp. L129
Source: Mar Drugs. 2019 Jun 26;17(7):383. doi: 10.3390/md17070383 (PMC6669556; doi:10.3390/md17070383)
Supplement: Supplementary file 1 [file marinedrugs-17-00383-s001.pdf]

Dimeric 1,4-benzoquinone Derivatives with Cytotoxic Activities from the Marine-Derived Fungus *Penicillium* sp. L129

Hui-Min Zhang [1], Chuan-Xia Ju [1], Gang Li [1], Yong Sun [2], Yu Peng [1], Ying-Xia Li [1] and Xiao-Ping Peng [1]*, and Hong-Xiang Lou [1,2]*

1 Department of Natural Medicinal Chemistry and Pharmacognosy, School of Pharmacy, Qingdao University, Qingdao 266021, China; 15725214505@163.com (H.-M.Z.); jucx@qdu.edu.cn (C.-X.J.); gang.li@qdu.edu.cn (G.L.); pengyupy@126.com (Y.P.); li_yx1964@163.com (Y.-X.L.)

2 Department of Natural Product Chemistry, Key Laboratory of Chemical Biology of Ministry of Education, School of Pharmaceutical Sciences, Shandong University, Jinan 250012, China; sunyongsy1@163.com

* Correspondence: louhongxiang@sdu.edu.cn (H.-X.L.); pengxiaoping@qdu.edu.cn (X.-P.P.); Tel.: +86-531-8838-2012 (H.-X.L.); +86-150-9229-3123 (X.-P.P.)

Abstract: Two new dimeric 1,4-benzoquinone derivatives, peniquinone A (**1**) and peniquinone B (**2**), a new dibenzofuran penizofuran A (**3**), and a new pyrazinoquinazoline derivative quinadoline D (**4**), together with 13 known compounds (**5-17**), were isolated from a marine-derived fungus *Penicillium* sp. L129. Their structures, including absolute configurations, were elucidated by extensive spectroscopic data and electronic circular dichroism calculations. Compound **1** exhibited cytotoxicity against the MCF-7, U87 and PC3 cell lines with IC_{50} values of 12.39 μM, 9.01 μM and 14.59 μM, respectively, while compound **2** displayed relatively weak cytotoxicity activities against MCF-7, U87 and PC3 cell lines with IC_{50} values of 25.32 μM, 13.45 μM and 19.93 μM, respectively. Furthermore, compound **2** showed weak quorum sensing inhibitory activity against *Chromobacterium violaceum* CV026 with an MIC value of 20 μg/well.

Keywords: marine-derived fungus; *Penicillium* sp.; secondary metabolites; quorum sensing; cytotoxicity; antifungal activity

Supplementary Information

Contents

Figure S1. The ^{1}H NMR (500 MHz, $CDCl_3$-*d*) spectrum of compound **1**

Figure S2. The ^{13}C NMR (125 MHz, $CDCl_3$-*d*) spectrum of compound **1**

Figure S3. The HSQC (500 MHz, $CDCl_3$-*d*) spectrum of compound **1**

Figure S4. The HMBC (500 MHz, $CDCl_3$-*d*) spectrum of compound **1**

Figure S5. The NOESY (500 MHz, $CDCl_3$-*d*) spectrum of compound **1**

Figure S6. The HRESIMS spectrum of compound **1**

Figure S7. IR spectrum of compound **1**

Figure S8. The ^{1}H NMR (500 MHz, DMSO-d_6) spectrum of compound **2**

Figure S9. The ^{13}C NMR (125 MHz, DMSO-d_6) spectrum of compound **2**

Figure S10. The HSQC (500 MHz, DMSO-d_6) spectrum of compound **2**

Figure S11. The HMBC (500 MHz, DMSO-d_6) spectrum of compound **2**

Figure S12. The ^{1}H-^{1}H COSY (500 MHz, DMSO-d_6) spectrum of compound **2**

Figure S13. The NOESY (500 MHz, DMSO-d_6) spectrum of compound **2**

Figure S14. The HRESIMS spectrum of compound **2**

Figure S15. IR spectrum of compound **2**

Figure S16. The ^{1}H NMR (500 MHz, DMSO-d_6) spectrum of compound **3**

Figure S17. The ^{13}C NMR (125 MHz, DMSO-d_6) spectrum of compound **3**

Figure S18. The HSQC (500 MHz, DMSO-d_6) spectrum of compound **3**

Figure S19. The HMBC (500 MHz, DMSO-d_6) spectrum of compound **3**

Figure S20. The NOESY (500 MHz, DMSO-d_6) spectrum of compound **3**

Figure S21. The HRESIMS spectrum of compound **3**

Figure S22. IR spectrum of compound **3**

Figure S23. The ^{1}H NMR (500 MHz, DMSO-d_6) spectrum of compound **4**

Figure S24. The ^{13}C NMR (125 MHz, DMSO-d_6) spectrum of compound **4**

Figure S25. The HSQC (500 MHz, DMSO-d_6) spectrum of compound **4**

Figure S26. The HMBC (500 MHz, DMSO-d_6) spectrum of compound **4**

Figure S27. The ^{1}H-^{1}H COSY (500 MHz, DMSO-d_6) spectrum of compound **4**

Figure S28. The NOESY (500 MHz, DMSO-d_6) spectrum of compound **4**

Figure S29. The NOE (500 MHz, DMSO-d_6) spectrum of compound **4**

Figure S30. The HRESIMS spectrum of compound **4**

Figure S31. IR spectrum of compound **4**

Figure S32. Computational details, the optimized conformers and equilibrium populations of compound **4**

Figure S33. The results of QS inhibitory activities of compounds **2** and **8**

Figure S34. The results of antifungal activities of compounds **12-15**

Figure S35. The 16S rDNA gene sequences data of *Penicillium* sp. L129

Figure S36. The HPLC data of crude extract of *Penicillium* sp. L129 and compounds **1-4**

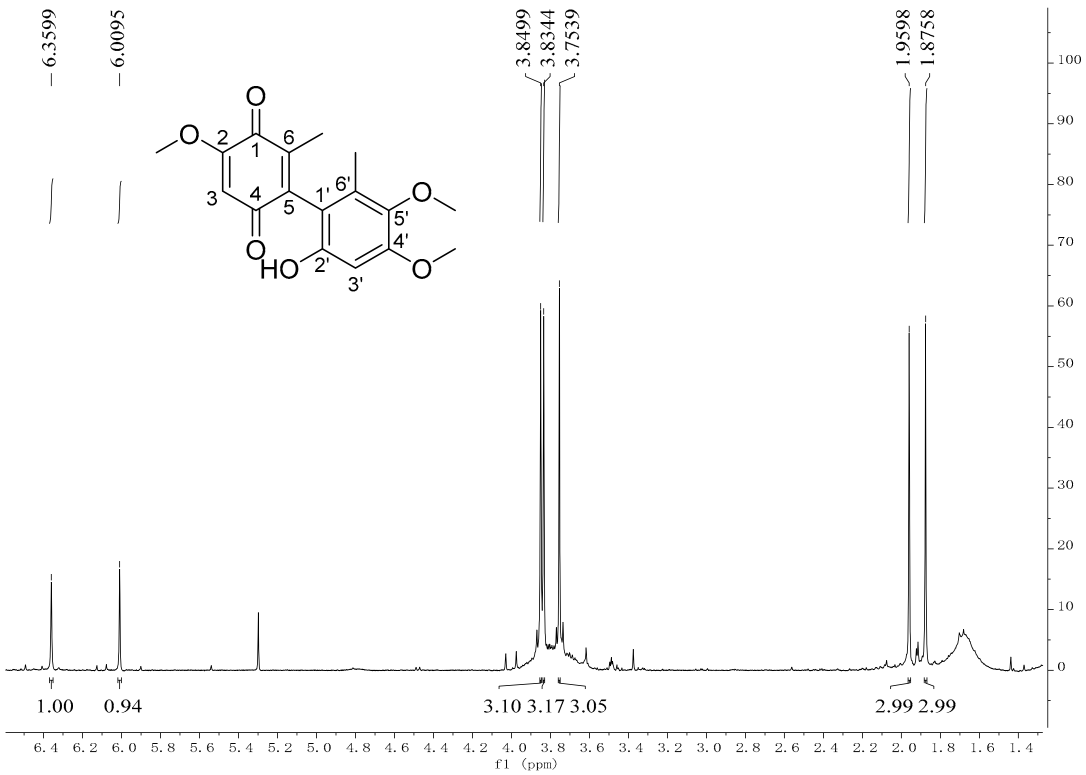

Figure S1. The ^{1}H NMR (500 MHz, $CDCl_3$-*d*) spectrum of compound **1**

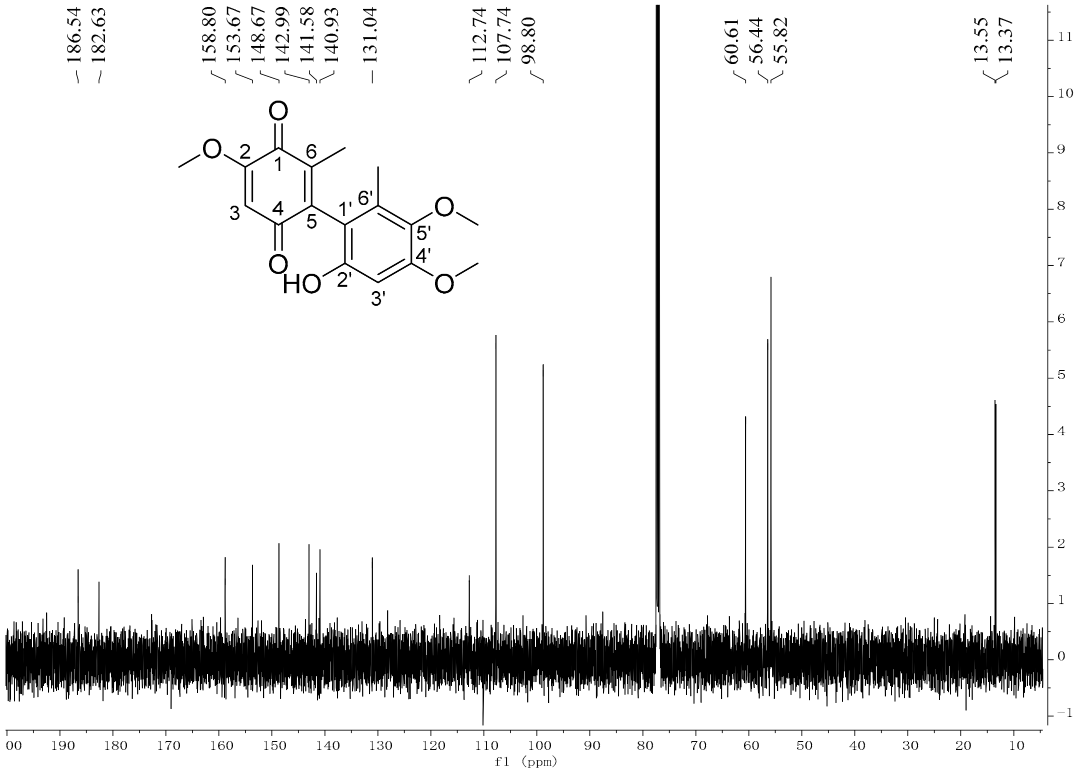

Figure S2. The ^{13}C NMR (125 MHz, $CDCl_3$-*d*) spectrum of compound **1**

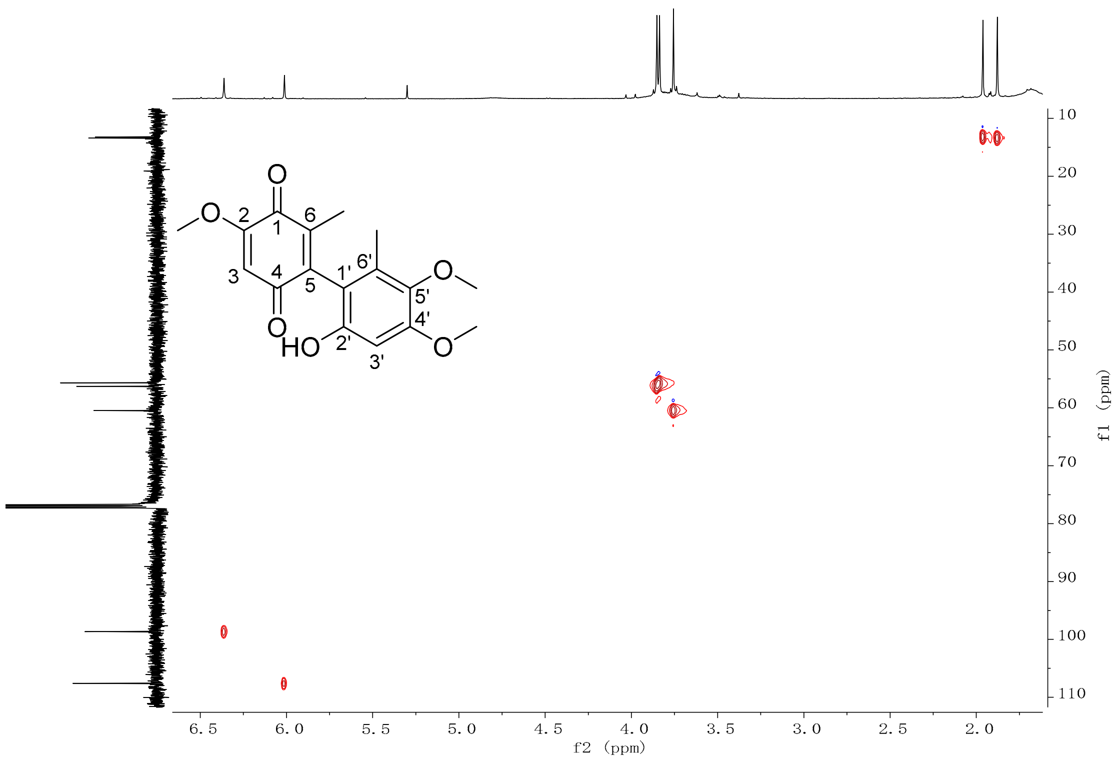

Figure S3. The HSQC (500 MHz, CDCl3-*d*) spectrum of compound **1**

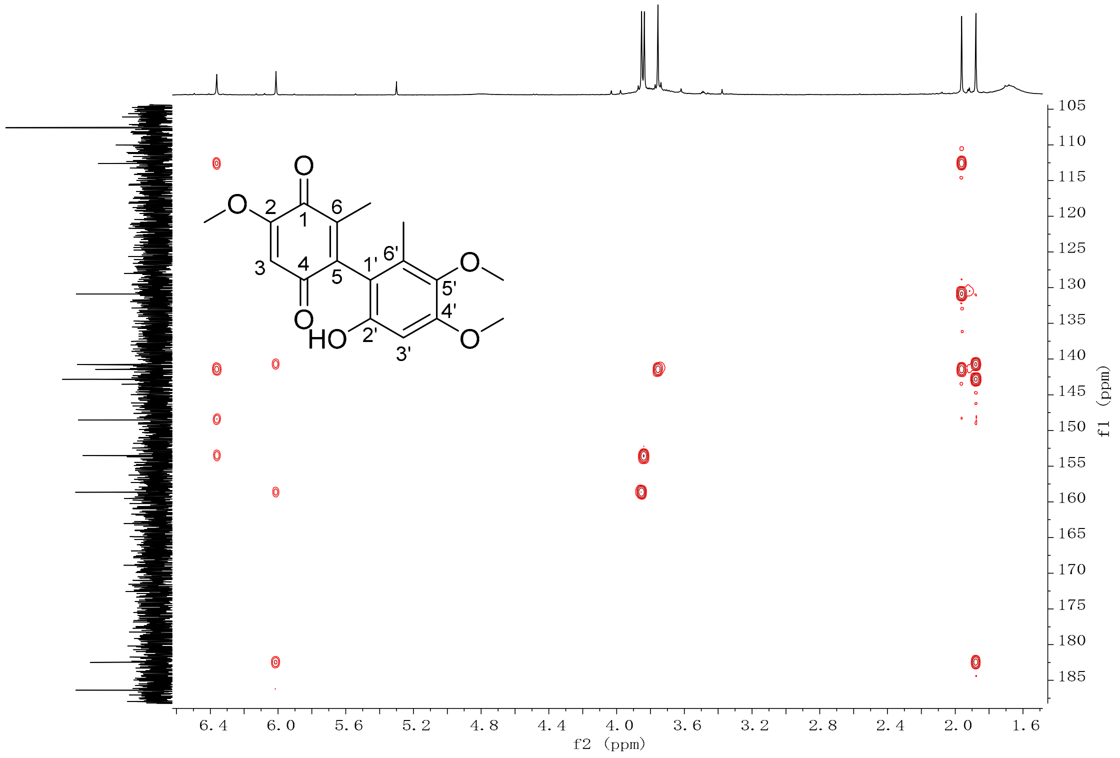

Figure S4. The HMBC (500 MHz, CDCl3-*d*) spectrum of compound **1**

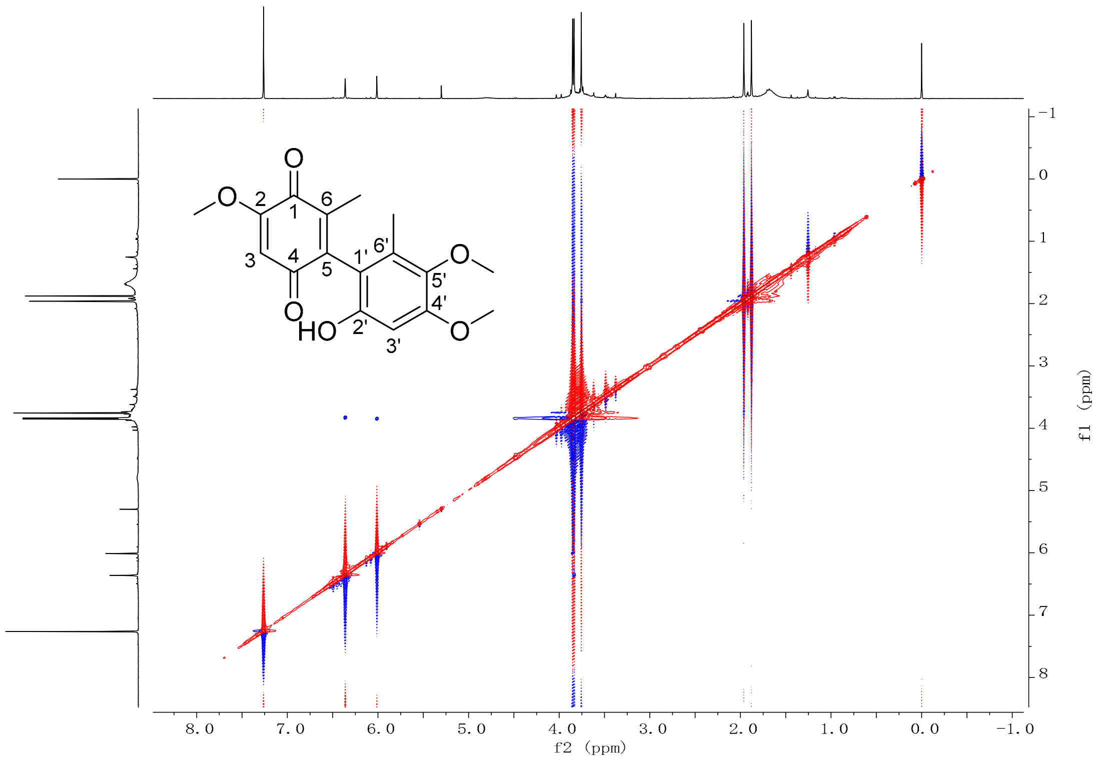

Figure S5. The NOESY (500 MHz, $CDCl_3$-*d*) spectrum of compound **1**

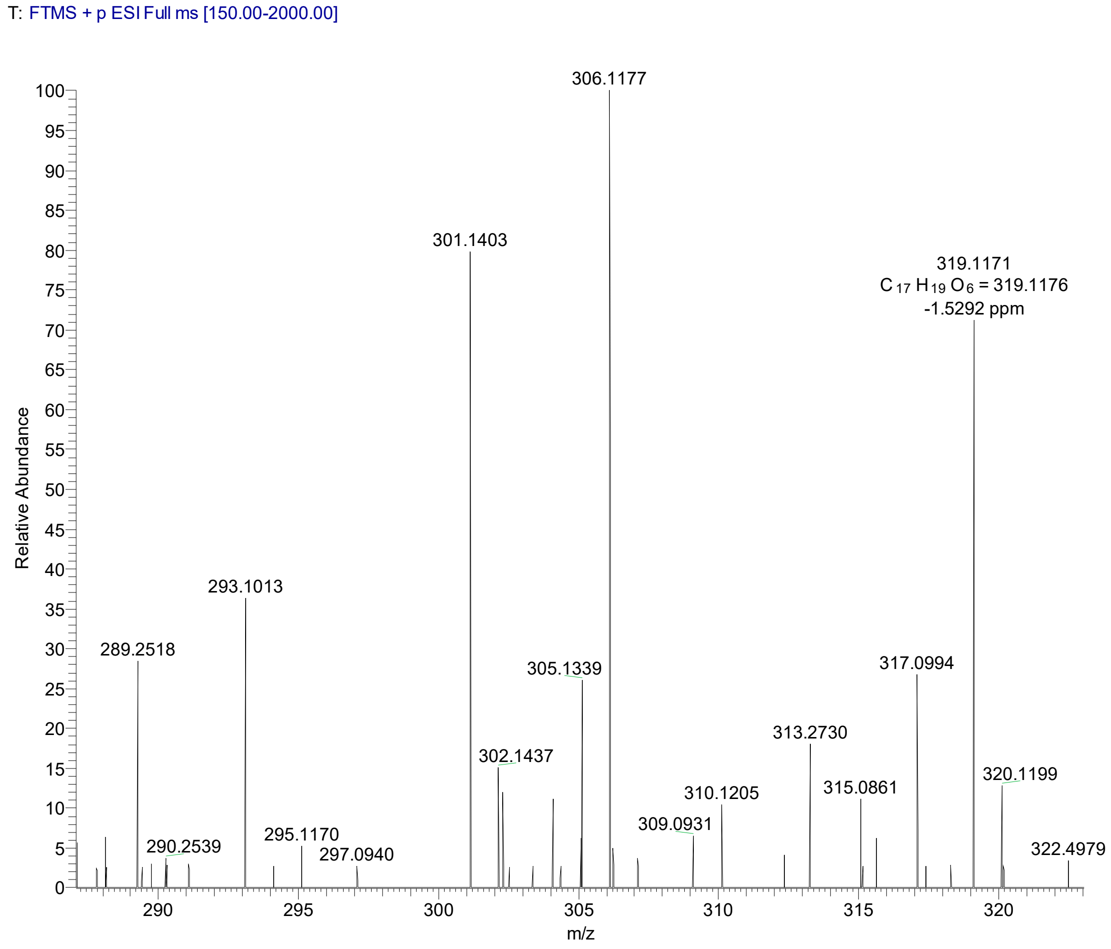

Figure S6. The HRESIMS spectrum of compound **1**

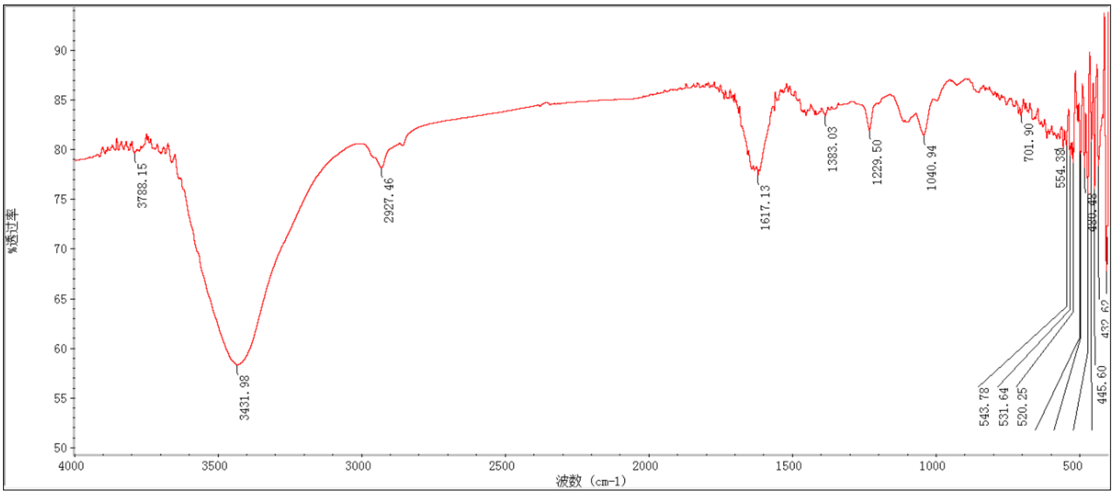

Figure S7. IR spectrum of compound **1**

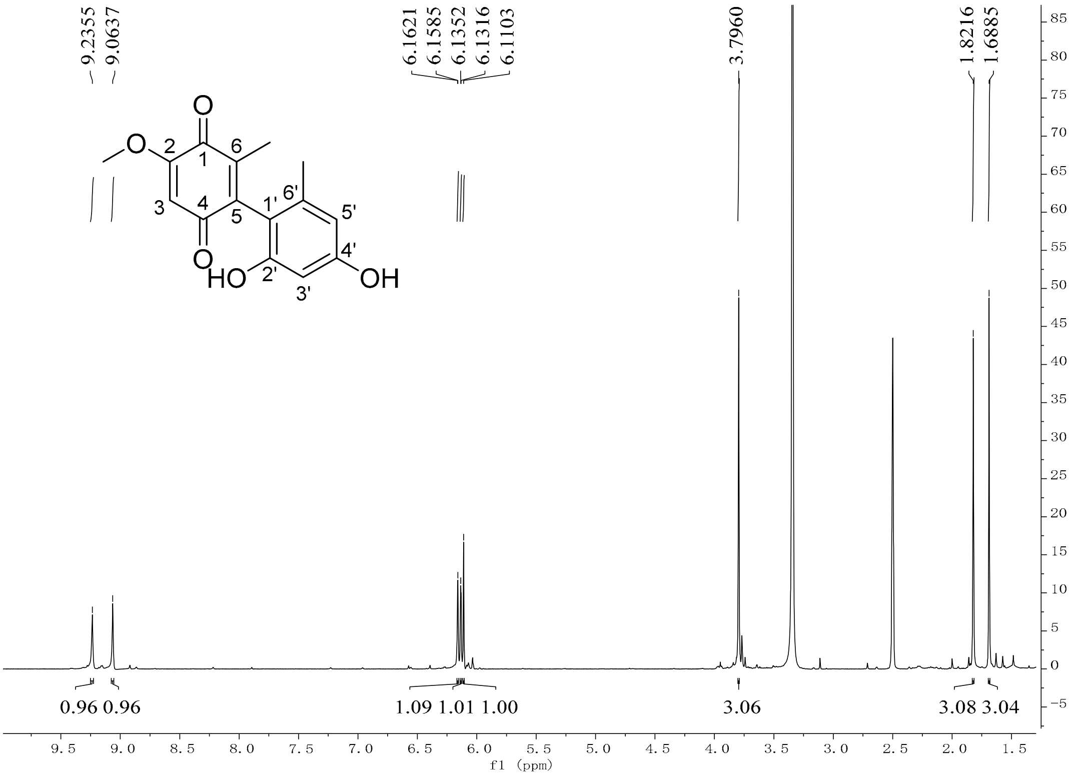

Figure S8. The ^{1}H NMR (500 MHz, DMSO-d_6) spectrum of compound **2**

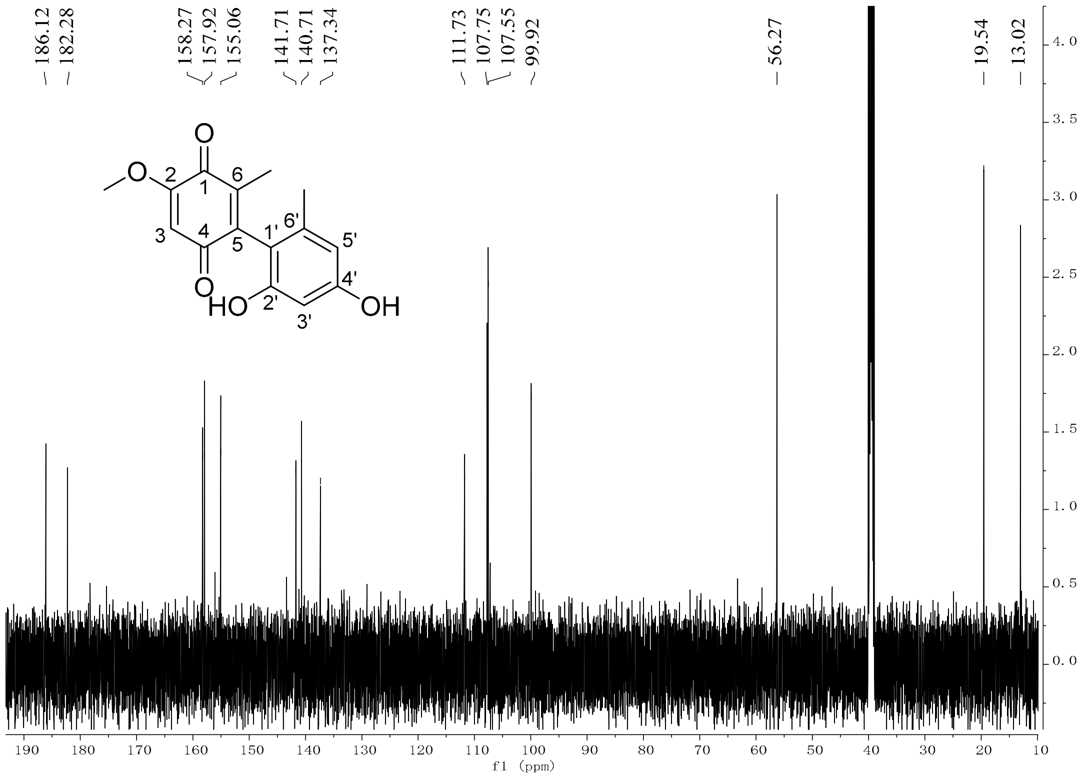

Figure S9. The ^{13}C NMR (125 MHz, DMSO-d_6) spectrum of compound **2**

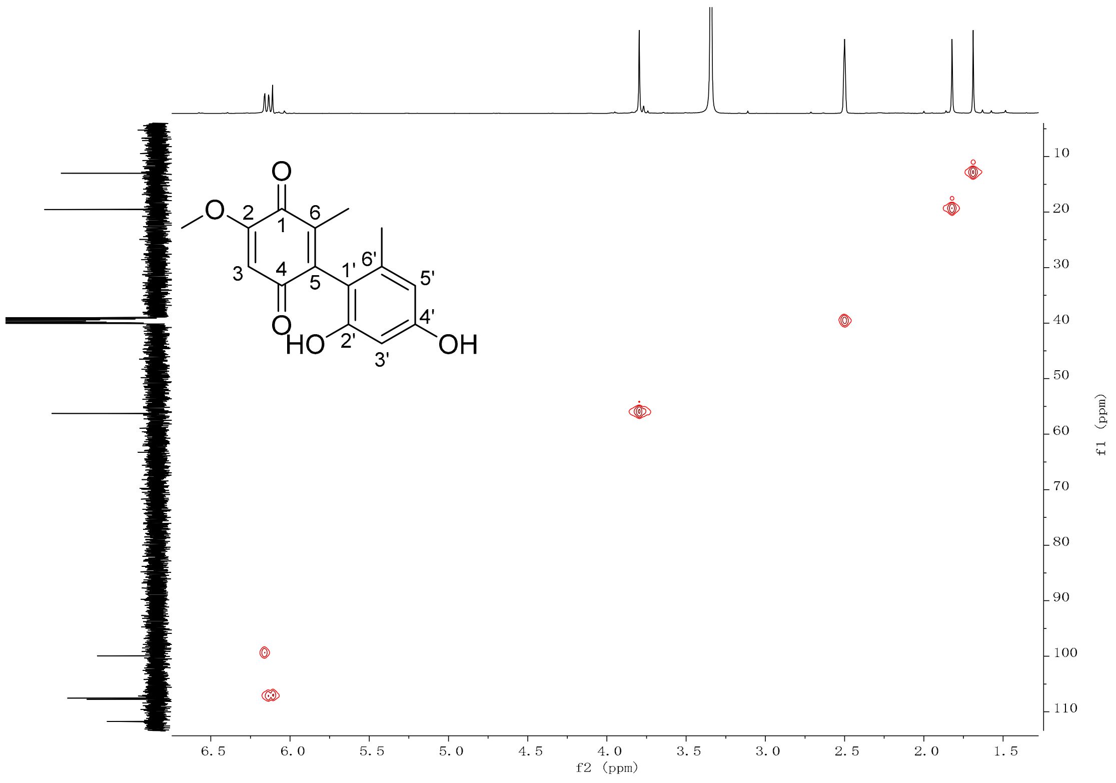

Figure S10. The HSQC (500 MHz, DMSO-d_6) spectrum of compound **2**

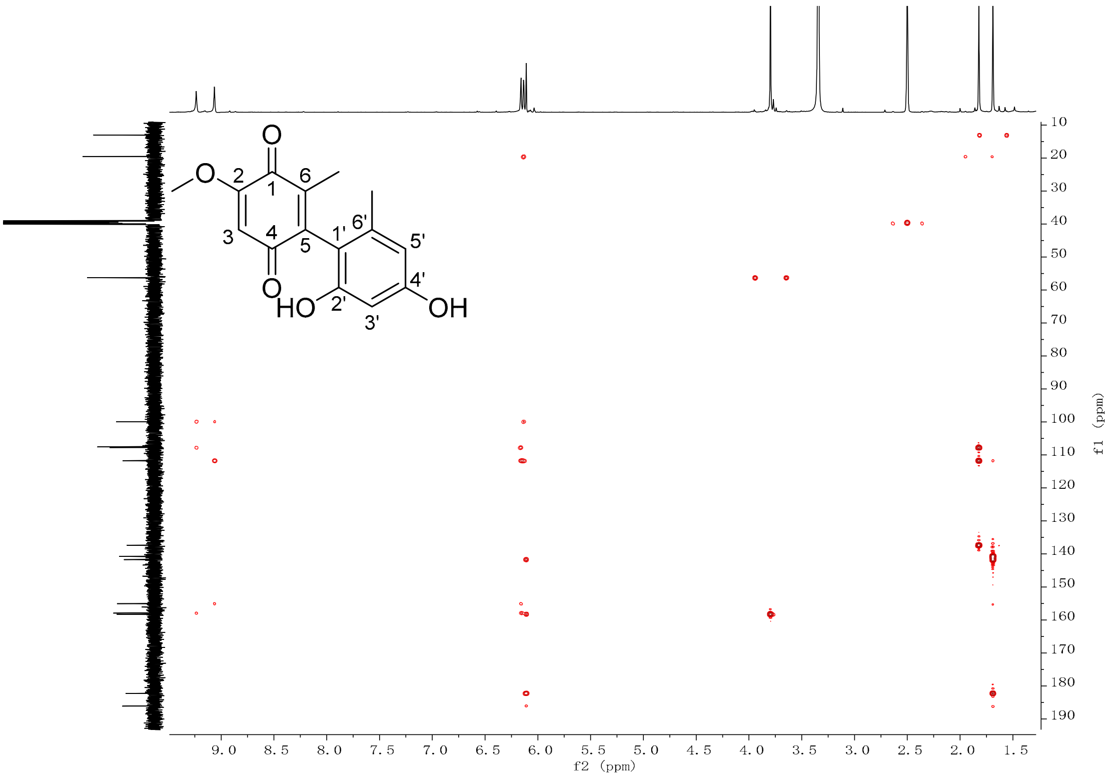

Figure S11. The HMBC (500 MHz, DMSO-d_6) spectrum of compound **2**

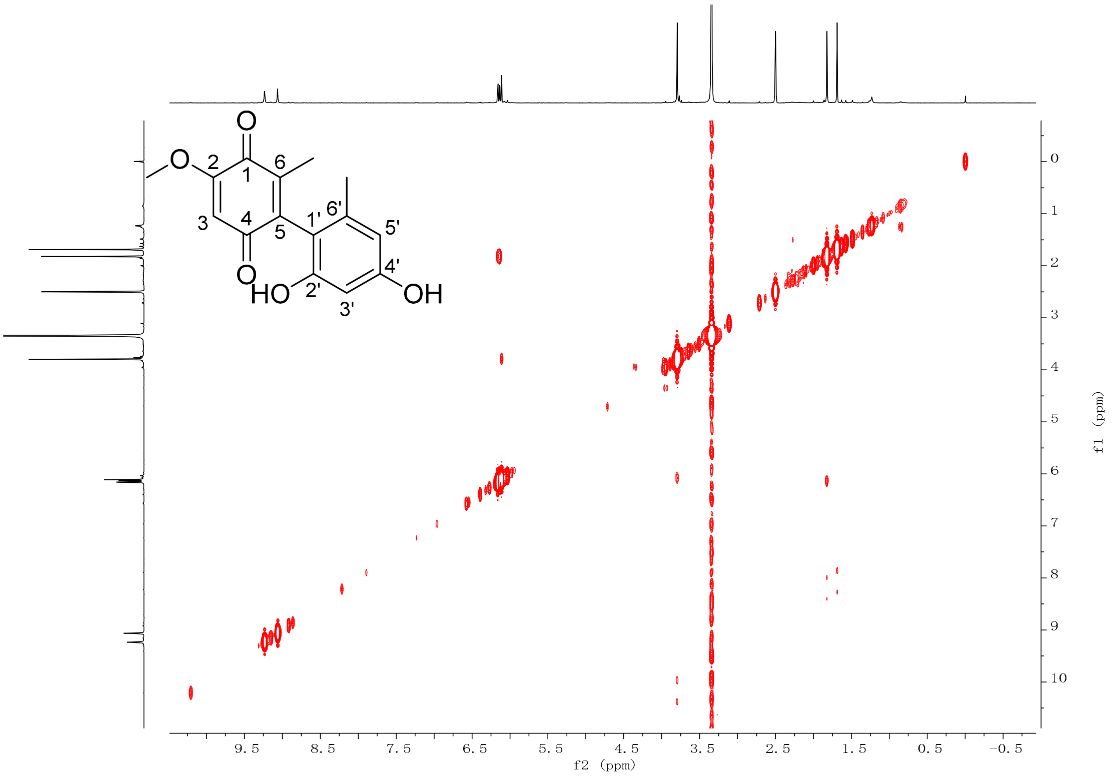

Figure S12. The ^{1}H-^{1}H COSY (500 MHz, DMSO-d_6) spectrum of compound **2**

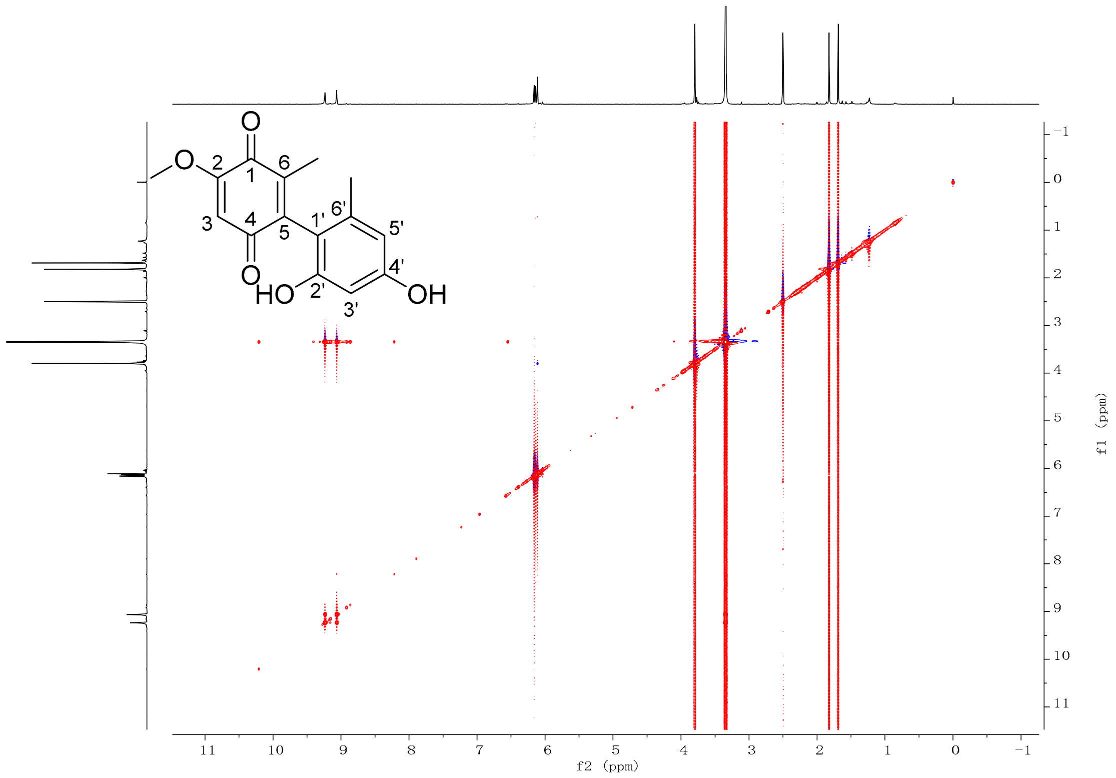

Figure S13. The NOESY (500 MHz, DMSO-d_6) spectrum of compound **2**

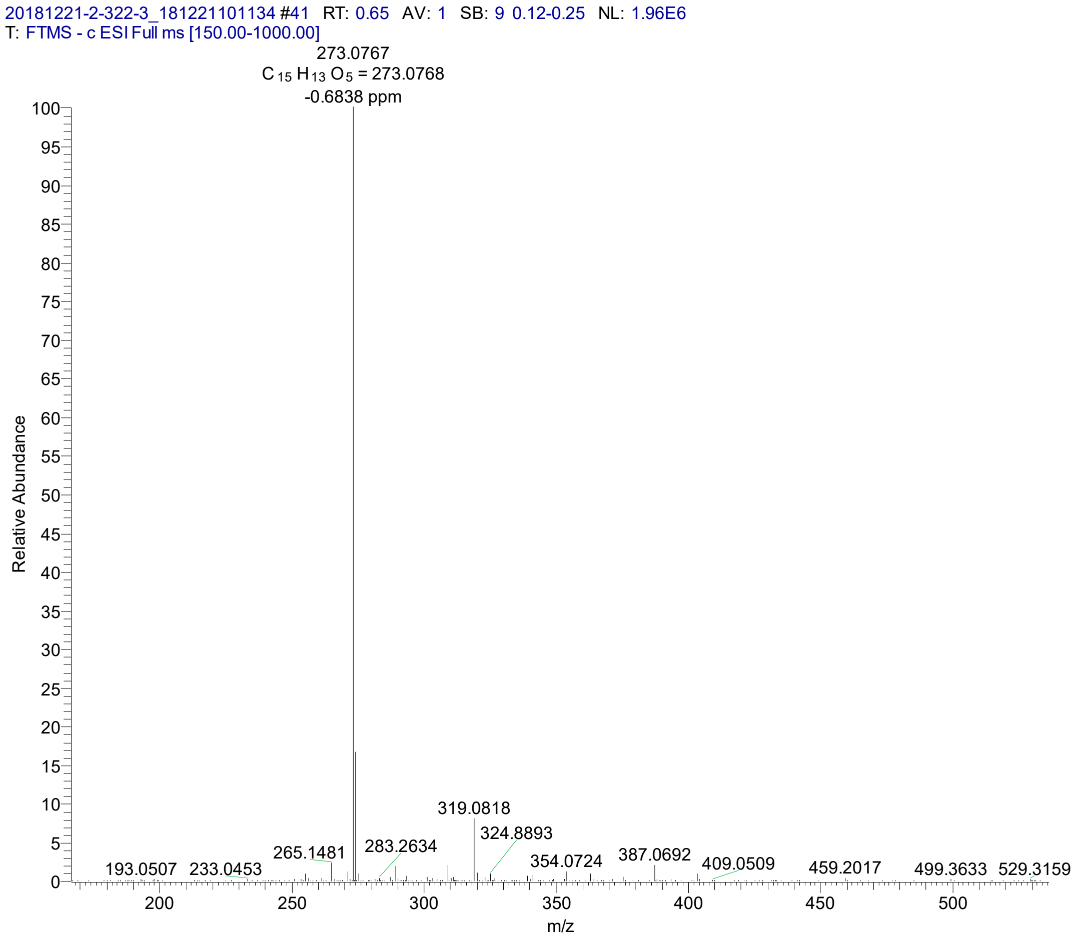

Figure S14. The HRESIMS spectrum of compound **2**

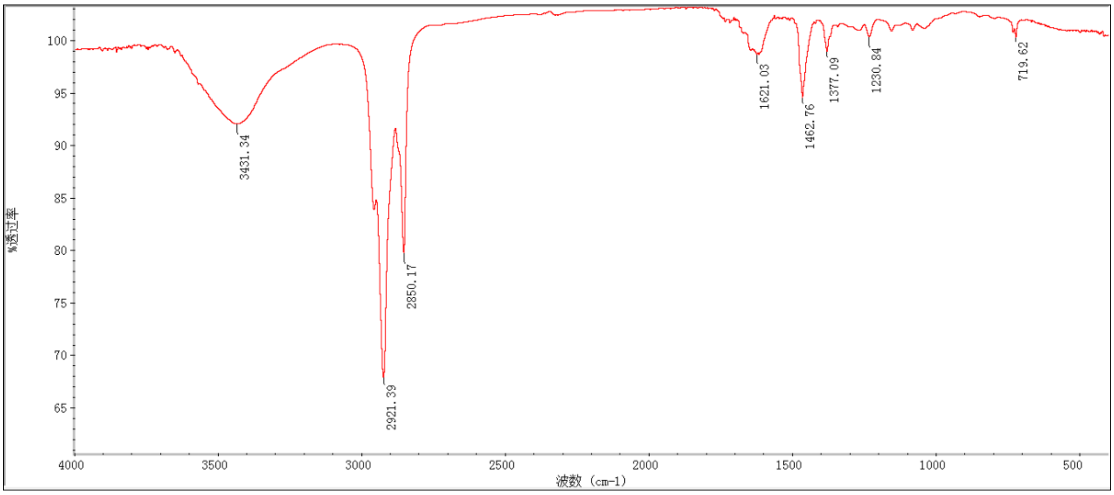

Figure S15. IR spectrum of compound **2**

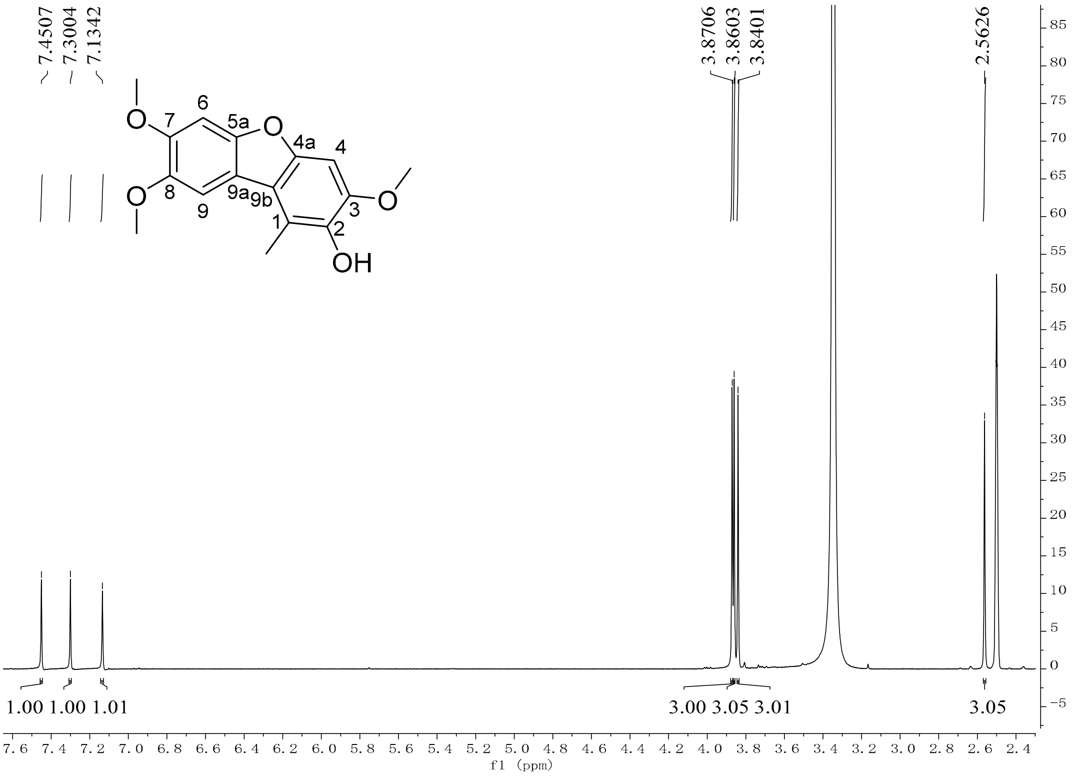

Figure S16. The ^{1}H NMR (500 MHz, DMSO-d_6) spectrum of compound **3**

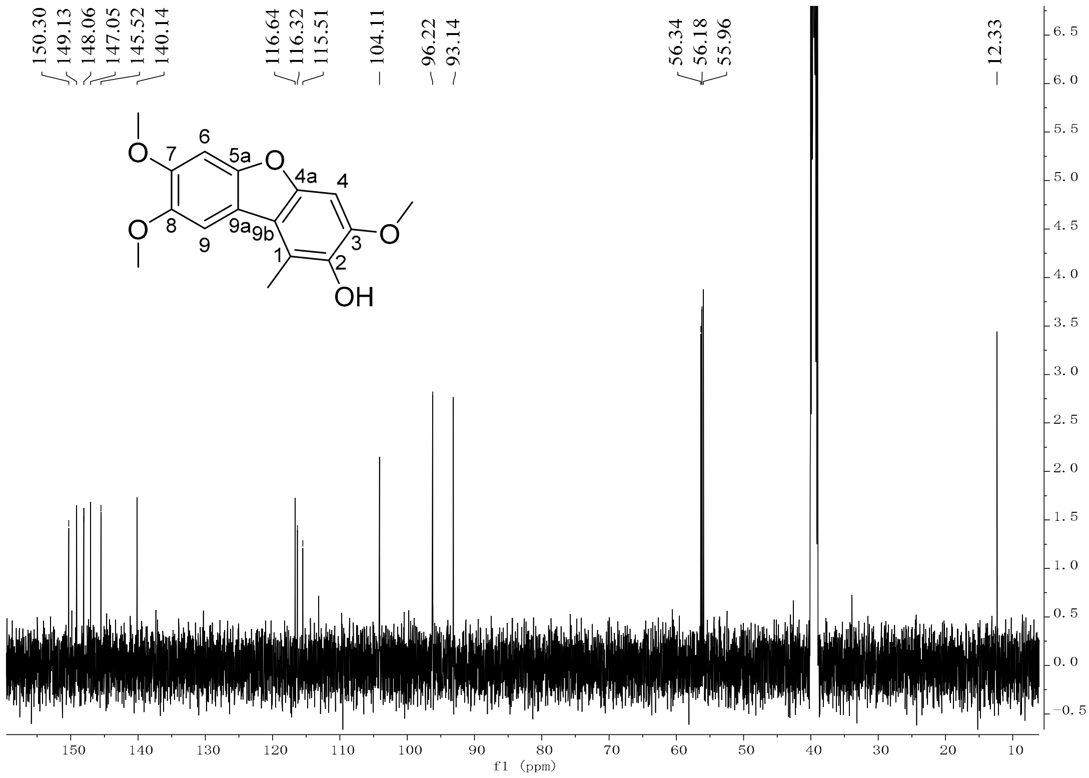

Figure S17. The ^{13}C NMR (125 MHz, DMSO-d_6) spectrum of compound **3**

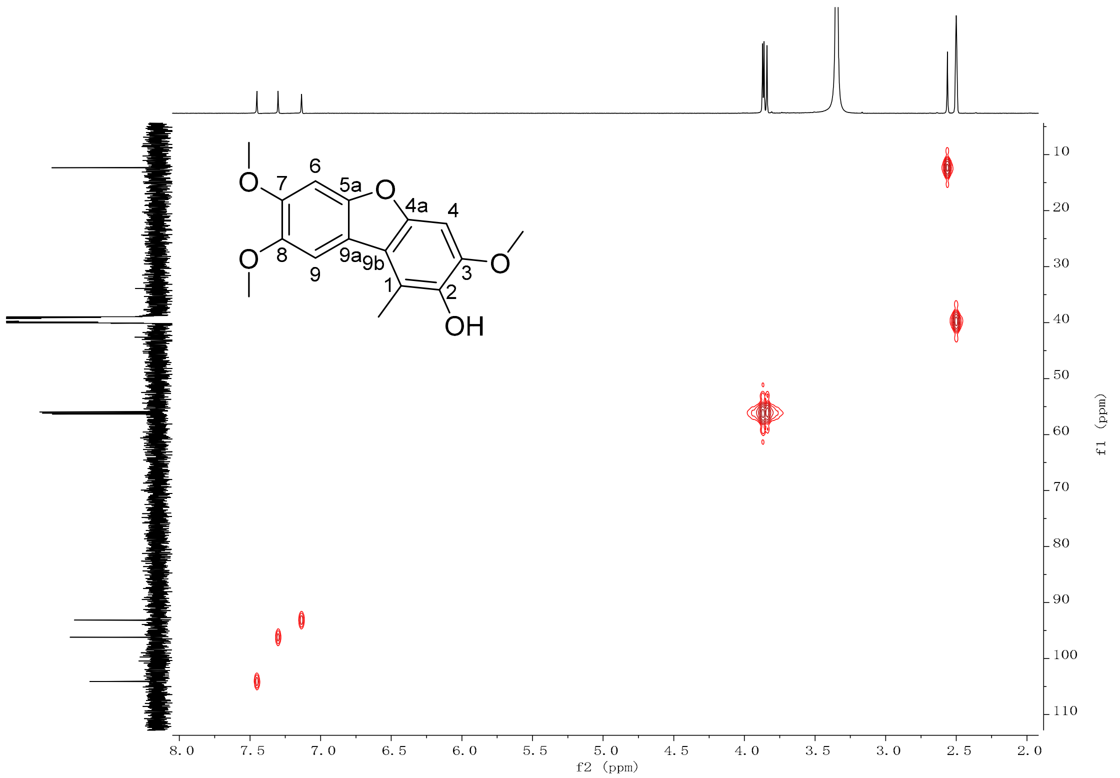

Figure S18. The HSQC (500 MHz, DMSO-d_6) spectrum of compound **3**

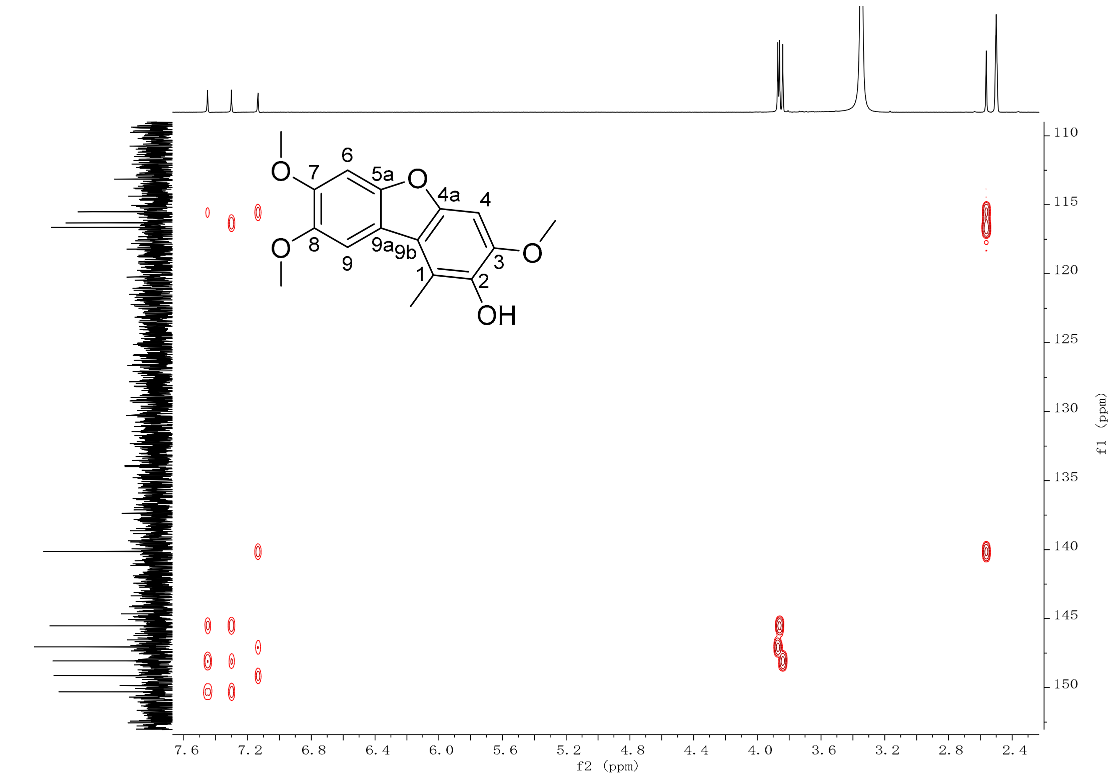

Figure S19. The HMBC (500 MHz, DMSO-d_6) spectrum of compound **3**

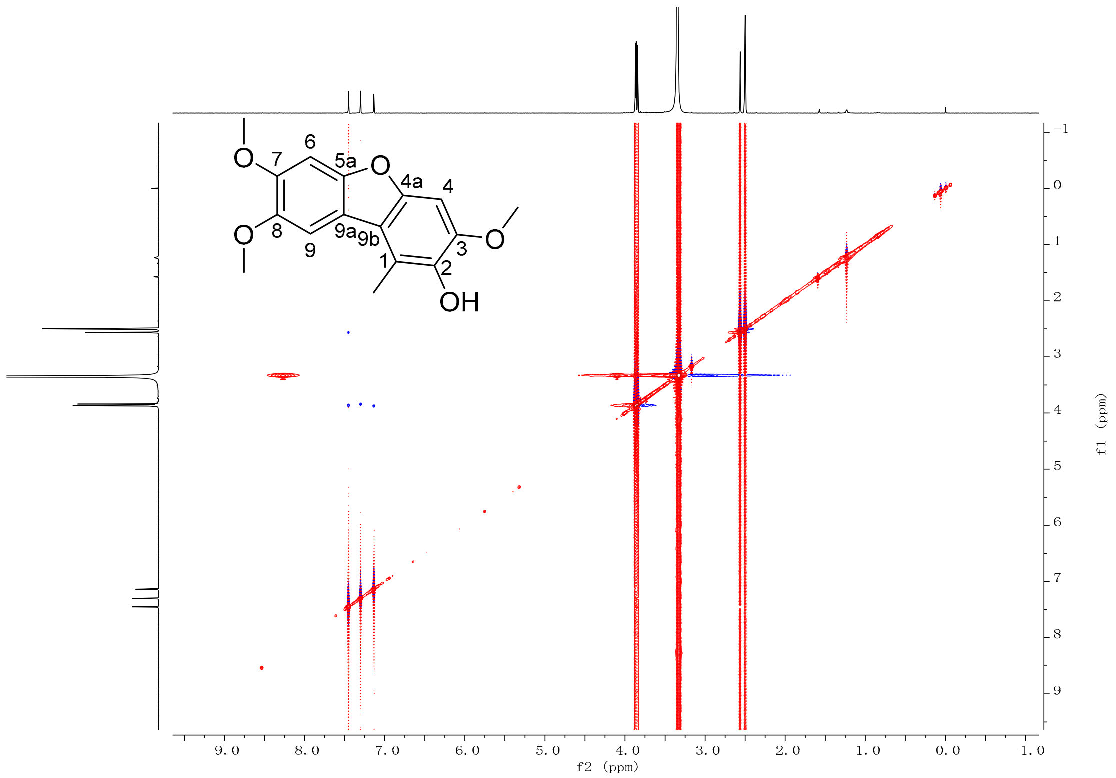

Figure S20. The NOESY (500 MHz, DMSO-d_6) spectrum of compound **3**

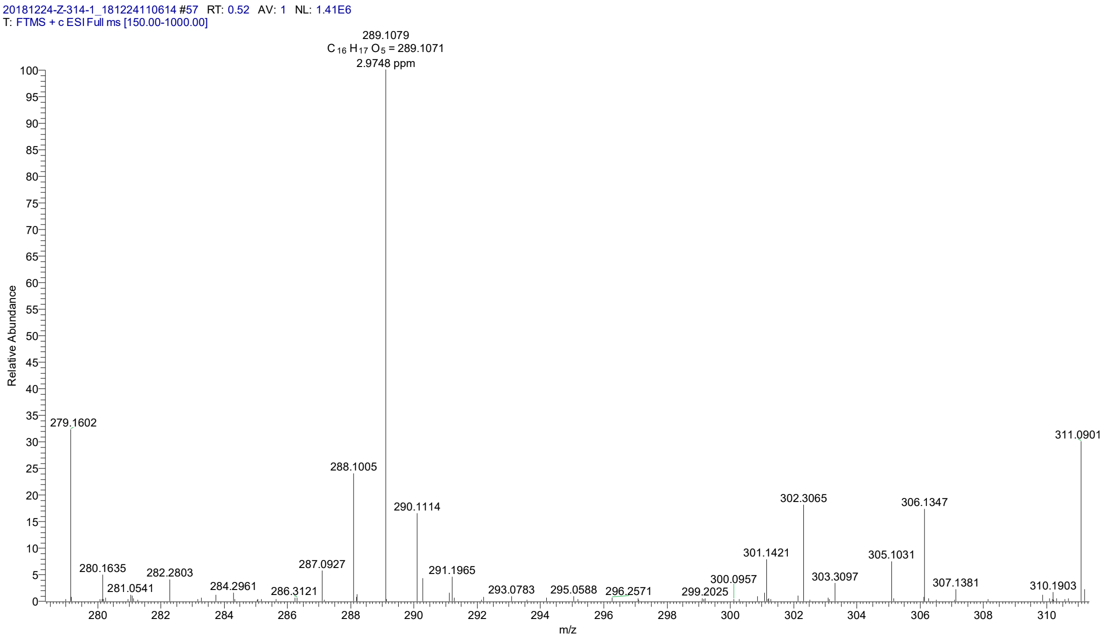

Figure S21. The HRESIMS spectrum of compound **3**

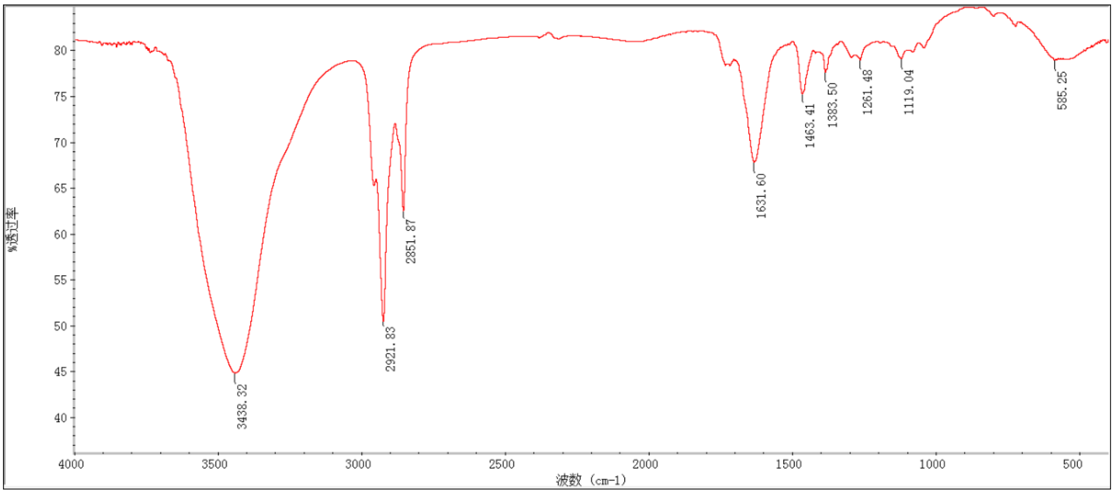

Figure S22. IR spectrum of compound **3**

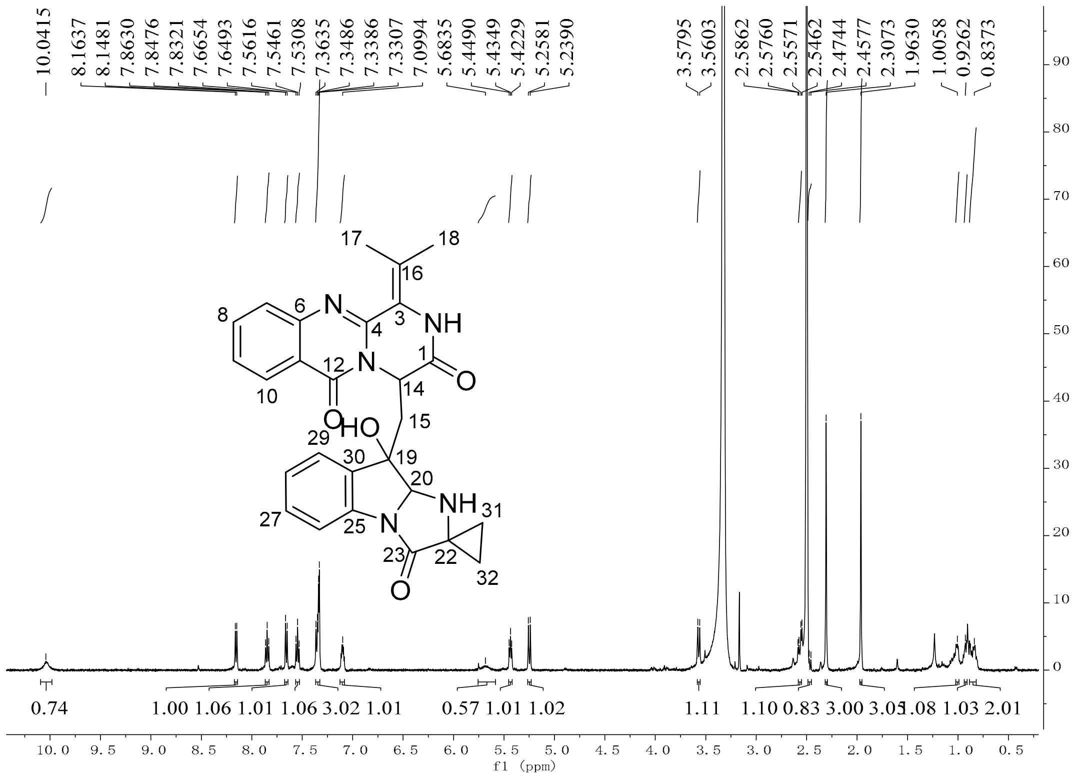

Figure S23. The ^{1}H NMR (500 MHz, DMSO-d_6) spectrum of compound **4**

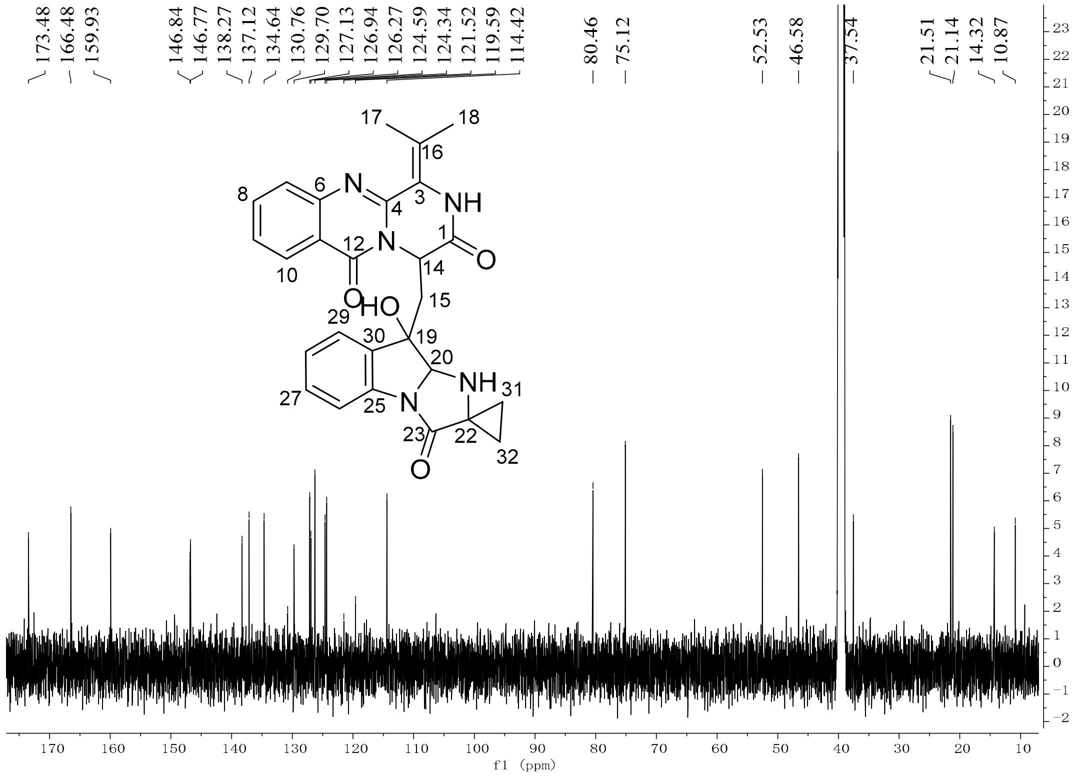

Figure S24. The ^{13}C NMR (125 MHz, DMSO-d_6) spectrum of compound **4**

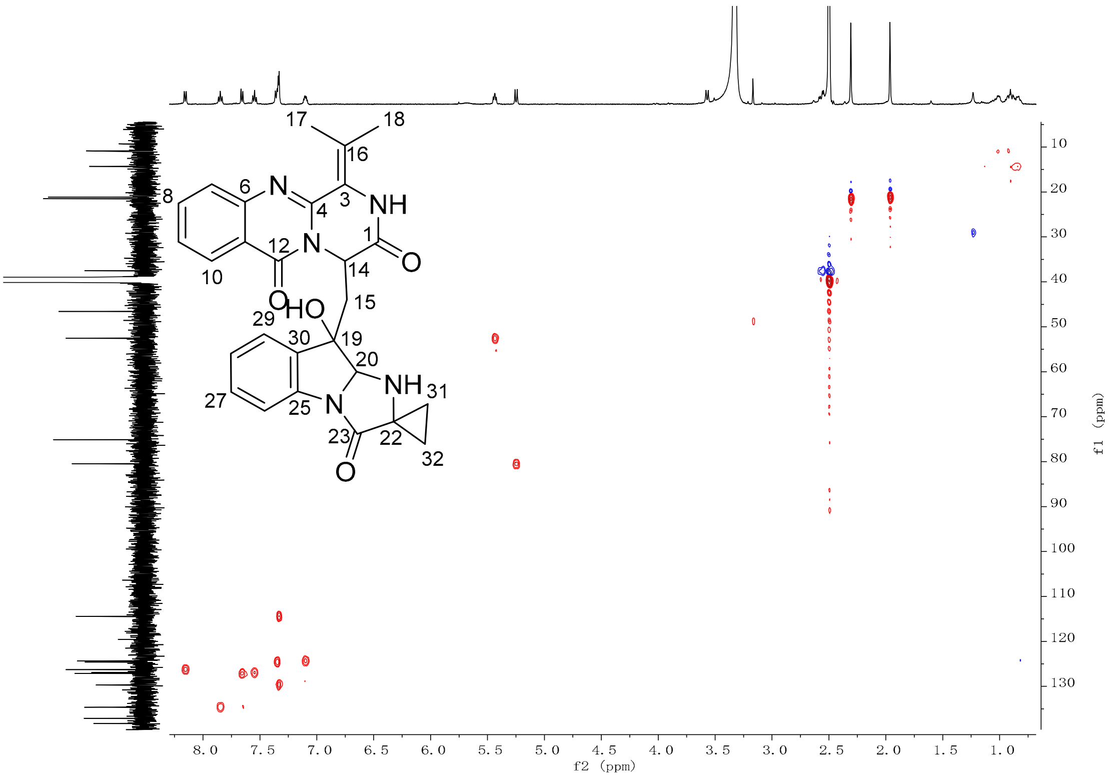

Figure S25. The HSQC (500 MHz, DMSO-d_6) spectrum of compound **4**

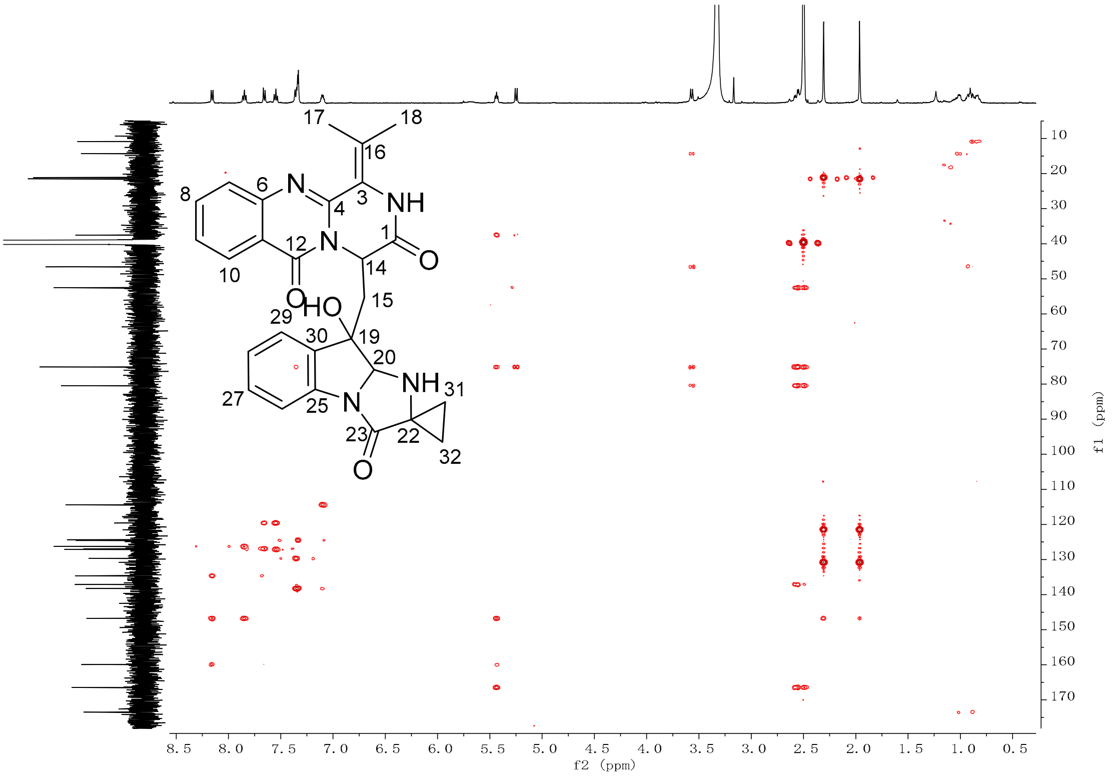

Figure S26. The HMBC (500 MHz, DMSO-d_6) spectrum of compound **4**

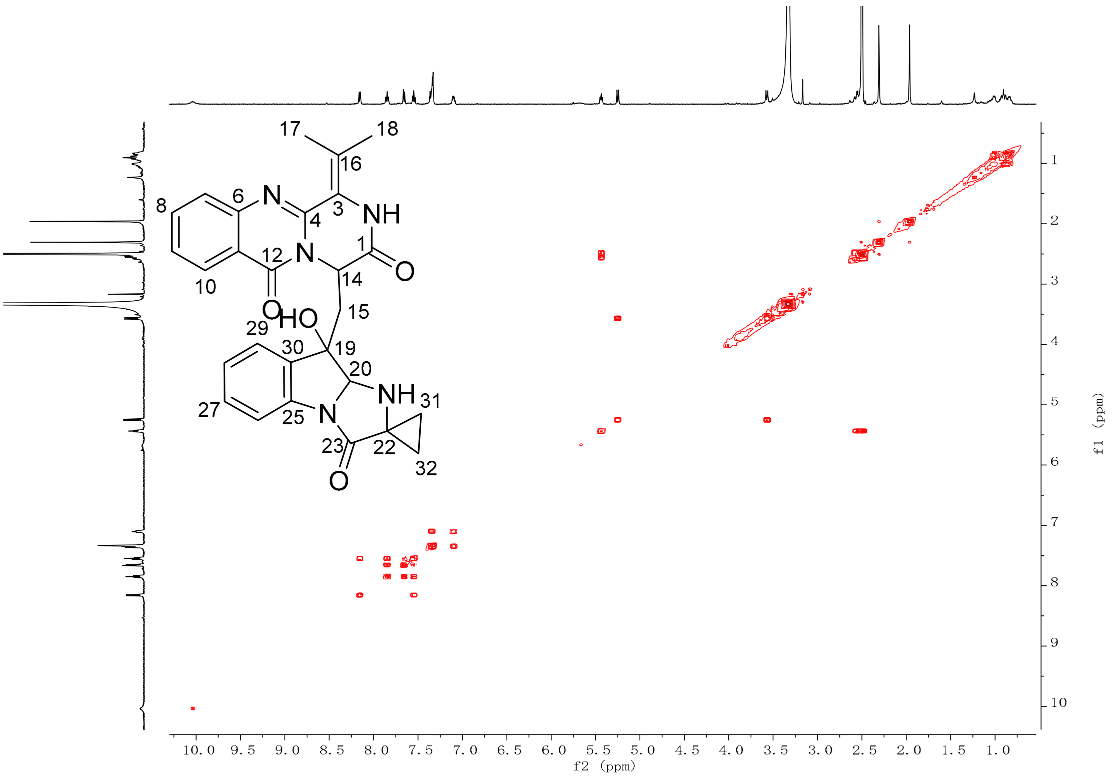

Figure S27. The ^{1}H-^{1}H COSY (500 MHz, DMSO-d_6) spectrum of compound **4**

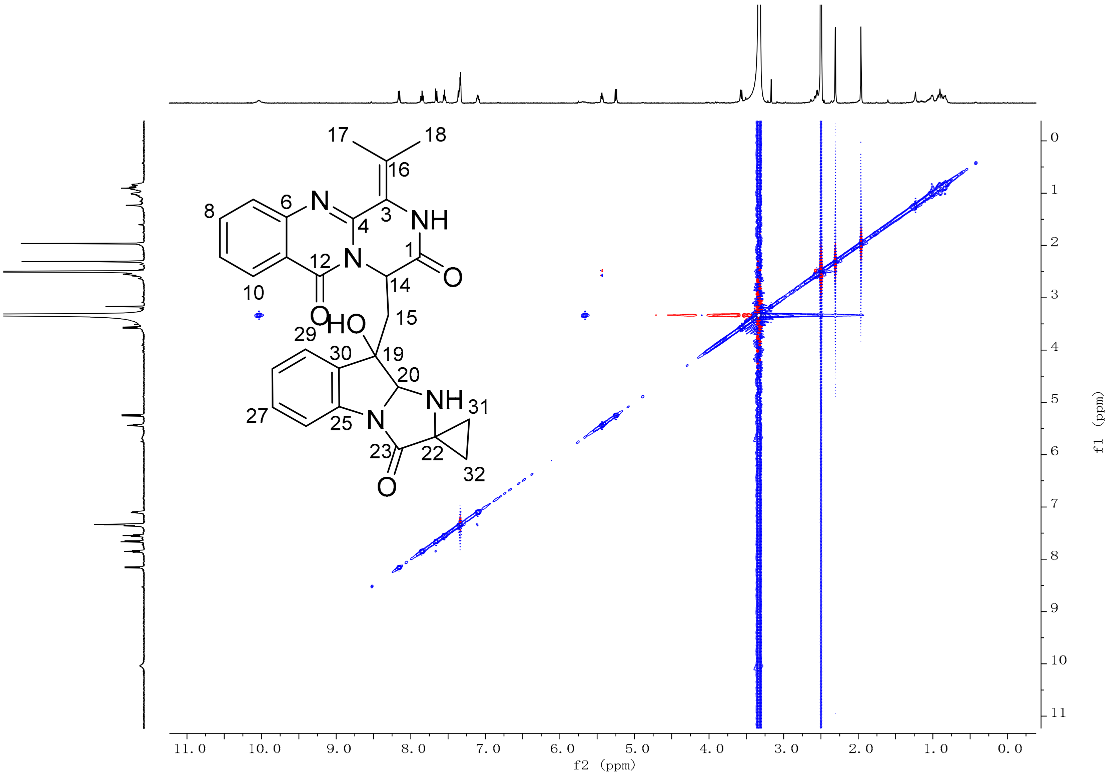

Figure S28. The NOESY (500 MHz, DMSO-d_6) spectrum of compound **4**

(because of low weight, the signals were meaningless, see NOE correlation, Figure S29)

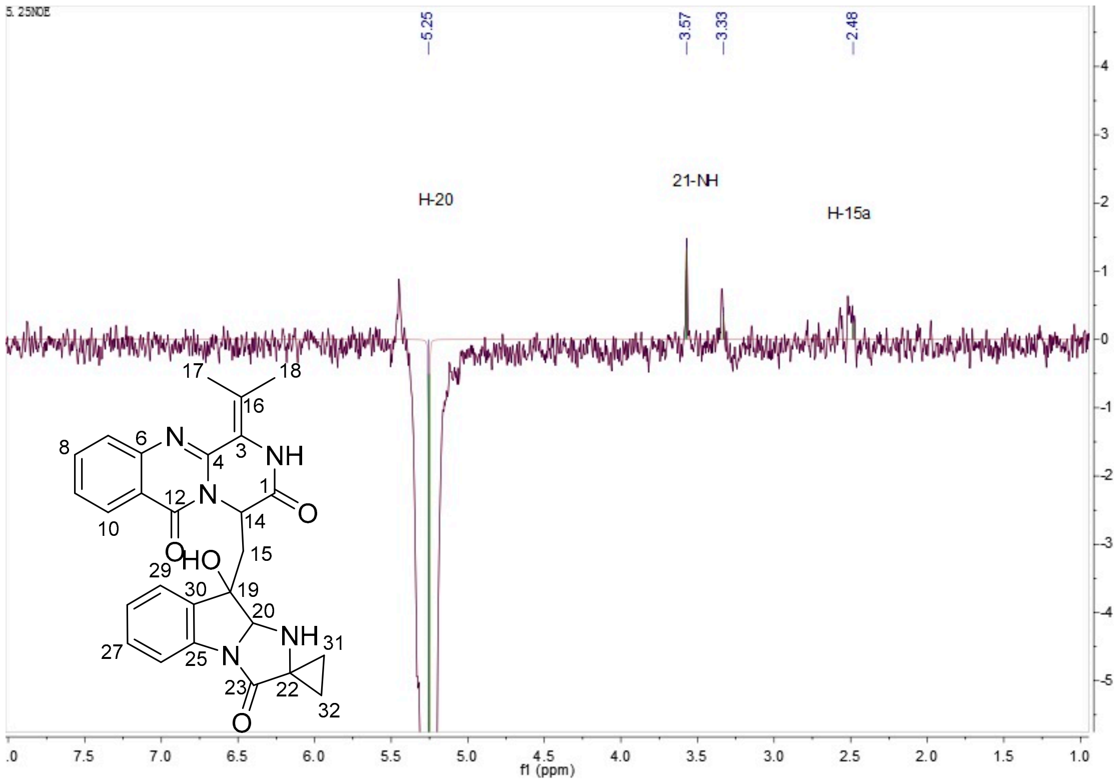

Figure S29. The NOE (500 MHz, DMSO-d_6) spectrum of compound **4**

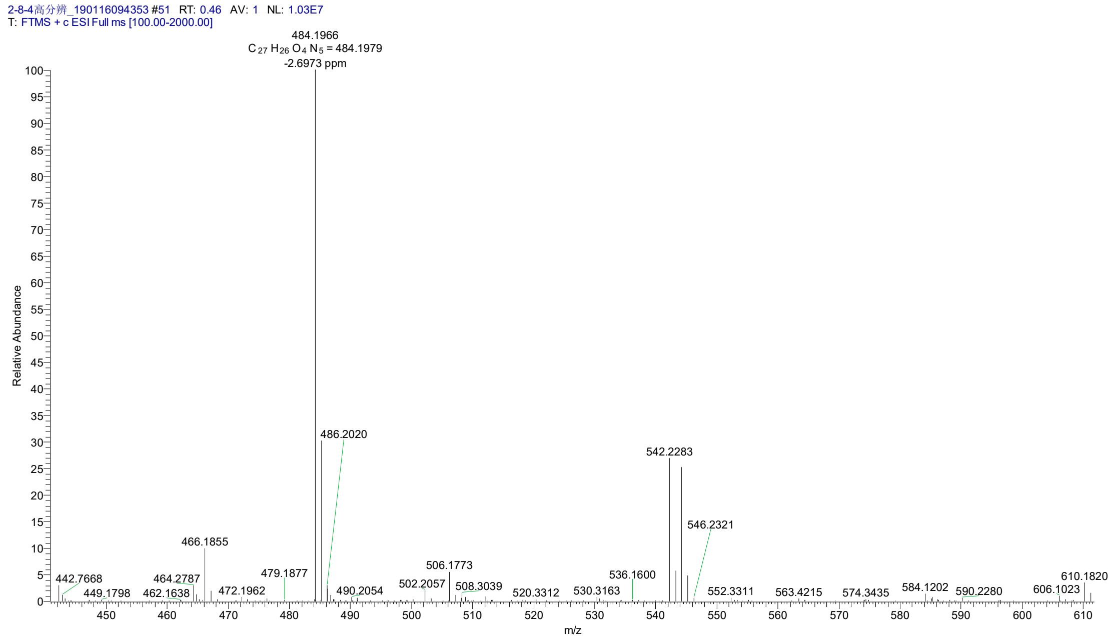

Figure S30. The HRESIMS spectrum of compound **4**

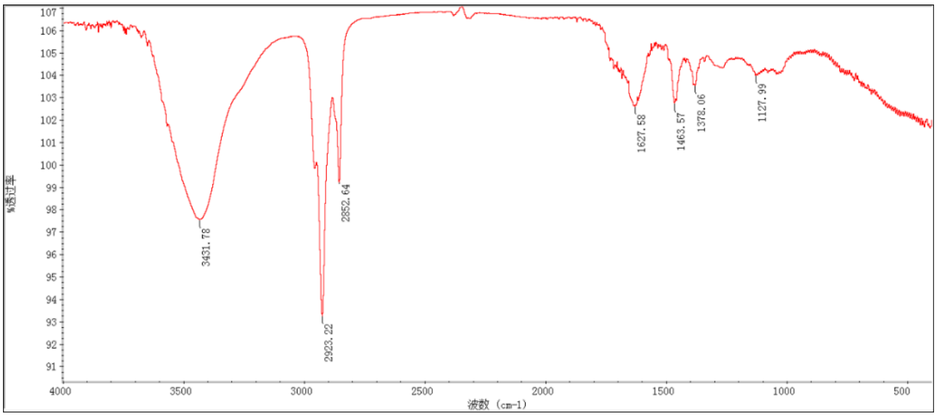

Figure S31. IR spectrum of compound **4**

ECD calculations for compound **4** were performed as describe in previous literature.[S1] The possible structural conformers were optimized using Frog2 online version.[S2] Further geometrical optimization and vibrational evaluation with the Gaussian g09 program were carried out, by using DFT calculations with the B3PW91 functional and the TZVP basis set. In order to calculate the singlet electronic excitation energies and rotational strengths with the solvation PCM model with methanol, TDDFT at the mPW1PW91/6-311G(d) level of theory was applied. The ECD curve for each conformer was then simulated according to equation 8d with a half-band of 0.3 eV.[S3] The individual conformer values were summed for obtaining the final ECD spectrum with respect to their Boltzmann distributions.

(14*R*, 19*S*, 20*R*)-**4**

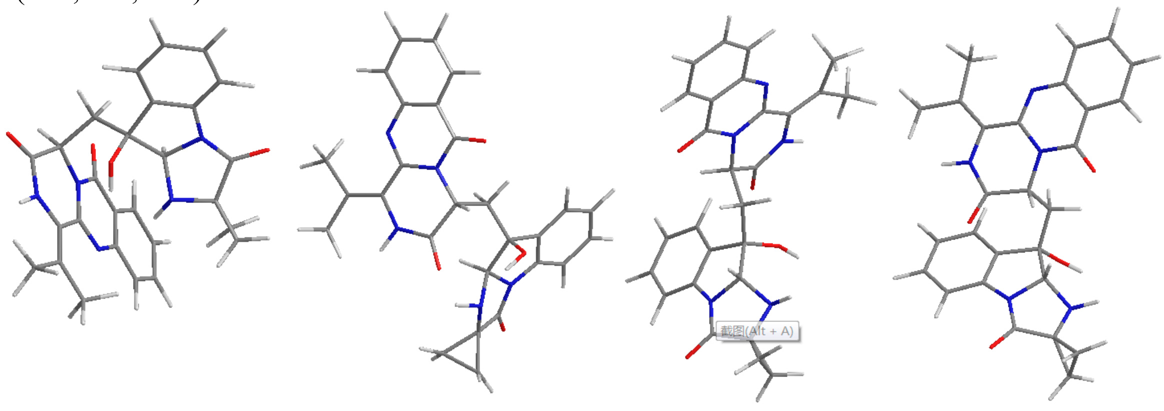

1 (21.02%) 2 (71.65%) 3 (0.54%) 4 (6.78)

Figure S32. Computational details**,** the optimized conformers and equilibrium populations of compound **4**

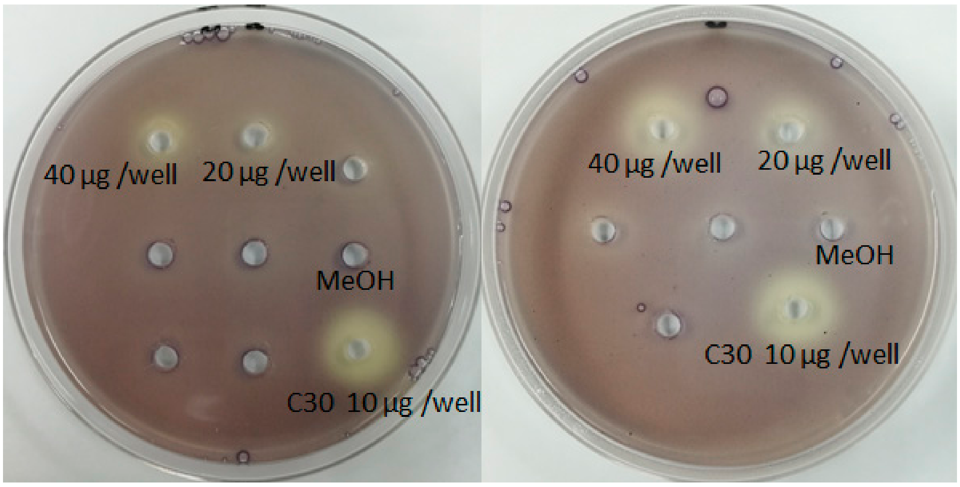

Compound **2** Compound **8**

Figure S33. The results of QS inhibitory activities of compounds **2** and **8**

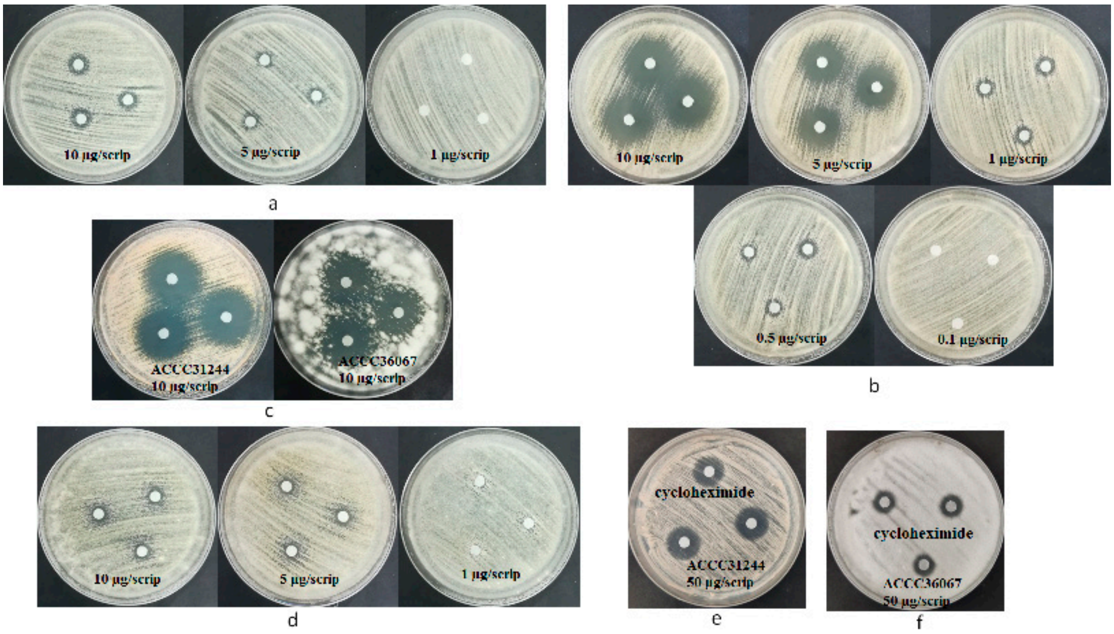

a: The serial solution of compound **12** against the fungus ACCC31244 (10, 5, 1 μg/scrip);

b: The serial solution of compound **13** against the fungus ACCC31244 (10, 5, 1,0.5, 0.1 μg/scrip);

c: The compound **14** against the fungus ACCC31244 and ACCC36067 (10 μg/scrip), compound **14** did not have enough mass to get MIC data;

d: The serial solution of compound **15** against the fungus ACCC31244 (10, 5, 1 μg/scrip);

e: Cycloheximide against the fungus ACCC31244 (50 μg/scrip);

f: Cycloheximide against the fungus ACCC36067 (50 μg/scrip)

Figure S34. The results of antifungal activities of compounds **12-15**

TAGGGTGACCTGCGGAAGGATCATTACCGAGCGAGAATTCTCTGAATTCAACCTCCCA
CCCGTGTTTATTGTACCTTGTTGCTTCGGCGGGCCCGCCTCACGGCCGCCGGGGGGCAT
CTGCCCCCGGGCCCGCGCCCGCCGAAGACACCTTGAACTCTGTATGAAAATTGCAGTC
TGAGTCTAAATATAAATTATTTAAAACTTTCAACAACGGATCTCTTGGTTCCGGCATCGA
TGAAGAACGCAGCGAAATGCGATACGTAATGTGAATTGCAGAATTCAGTGAATCATCG
AGTCTTTGAACGCACATTGCGCCCCCTGGTATTCCGGGGGGCATGCCTGTCCGAGCGT
CATTGCTGCCCTCAAGCCCGGCTTGTGTGTTGGGTCTCGTCCCCCTTCCCGGGGGGAC
GGGCCCGAAAGGCAGCGGCGGCACCGCGTCCGGTCCTCGAGCGTATGGGGCTTTGTC
ACCCGCTCTGTAGGCCCGGCCGGCGCTTGCCGATCAACCAAACCTTTTTCCAGGTTG
ACCTCGGATCAGGTAGGGATACCCGCTGAACTTAAGCATATCAATAAGCGGAG

Figure S35. The 16S rDNA gene sequences data of *Penicillium* sp. L129

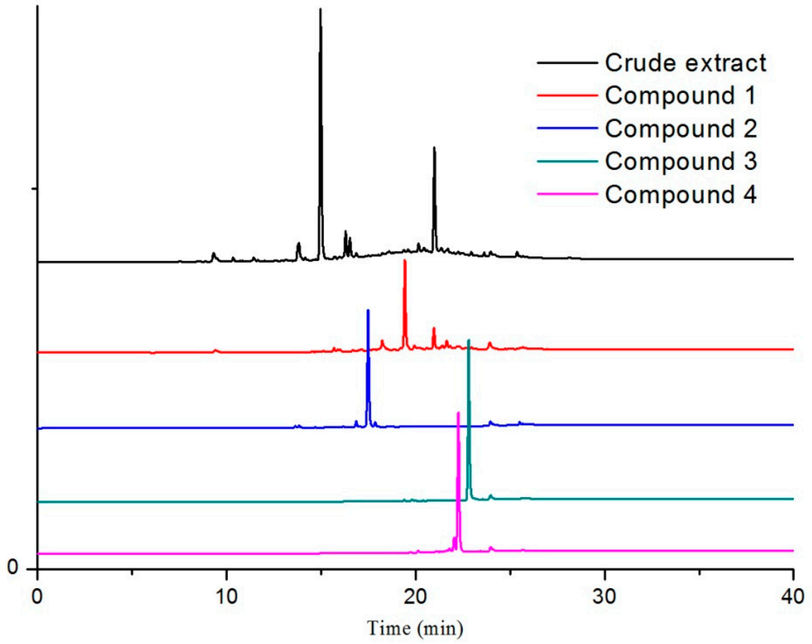

Due to the small mass, each of the compounds **1**-**4**, which was obtained from *Penicillium* sp. L129, showed insignificant peak in the HPLC data of crude extract. (crude extract: 40.7 g, compound **1**: 2.0 mg, compound **2**: 4.8 mg, compound **3**: 1.7 mg, compound **4**: 1.7 mg)

Figure S36. The HPLC data of crude extract of *Penicillium* sp. L129 and compounds **1-4** (MeOH/H_2O, 0-20 min 10-100% MeOH, 20-35 min, 100% MeOH, 35-40 min, 10% MeOH, UV-254 nm)

References

(S1) Wang, Z.R.; Li, G.; Ji, L.X.; Wang, H.H.; Gao, H.; Peng. X.P.; Lou, H.X. Induced production of steroids by co-cultivation of two endophytes from *Mahonia fortunei. Steroids* **2019**, *145*, 1–4.

(S2) Miteva, M.A.; Guyon, F.; Tuffery, P. Frog2: Efficient 3D conformation ensemble generator for small compounds. *Nucleic Acids Res.* **2010**, *38*, W622–627.

(S3) Stephens, P.J.; Harada, N. ECD Cotton Effect Approximated by the Gaussian Curve and Other Methods ECD Cotton Effect Approximated by the Gaussian Curve and Other Methods. *Chirality* **2010**, *22*, 229–233.
